# The Prevalence and Drug Sensitivity of Tuberculosis among Patients Dying in Hospital in KwaZulu-Natal, South Africa: A Postmortem Study

**DOI:** 10.1371/journal.pmed.1000296

**Published:** 2010-06-22

**Authors:** Ted Cohen, Megan Murray, Kristina Wallengren, Gonzalo G. Alvarez, Elizabeth Y. Samuel, Douglas Wilson

**Affiliations:** 1 Division of Global Health Equity, Brigham and Women's Hospital, Boston, Massachusetts, United States of America; 2 Department of Epidemiology, Harvard School of Public Health, Boston, Massachusetts, United States of America; 3 Infectious Disease Unit, Massachusetts General Hospital, Boston, Massachusetts, United States of America; 4 Department of Medicine, University of Ottawa at The Ottawa Hospital, Ottawa, Ontario, Canada; 5 National Health Laboratory Service, Inkosi Albert Luthuli Central Hospital, Mayville, South Africa; 6 Department of Medicine, Edendale Hospital, Pietermaritzburg, University of KwaZulu-Natal, South Africa; Perinatal HIV Research Unit, South Africa

## Abstract

**Background:**

Tuberculosis is the leading cause of death in South Africa by death notification, but accurate diagnosis of tuberculosis is challenging in this setting of high HIV prevalence. We conducted limited autopsies on young adults dying in a single public hospital in the province of KwaZulu-Natal between October 2008 and August 2009 in order to estimate the magnitude of deaths attributable to tuberculosis.

**Methods and Findings:**

We studied a representative sample of 240 adult inpatients (aged 20–45 years) dying after admission to Edendale Hospital. Limited autopsies included collection of respiratory tract secretions and tissue by needle core biopsies of lung, liver, and spleen. Specimens were examined by fluorescent microscopy for acid-fast bacilli and cultured in liquid media; cultures positive for *M. tuberculosis* were tested for drug susceptibility to first- and second-line antibiotics. Ninety-four percent of our study cohort was HIV seropositive and 50% of decedents had culture-positive tuberculosis at the time of death. Fifty percent of the participants were on treatment for tuberculosis at the time of death and 58% of these treated individuals remained culture positive at the time of death. Of the 50% not receiving tuberculosis treatment, 42% were culture positive. Seventeen percent of all positive cultures were resistant to both isoniazid and rifampin (i.e., multidrug resistant); 16% of patients dying during the initiation phase of their first ever course of tuberculosis treatment were infected with multidrug-resistant bacilli.

**Conclusions:**

Our findings reveal the immense toll of tuberculosis among HIV-positive individuals in KwaZulu-Natal. The majority of decedents who remained culture positive despite receiving tuberculosis treatment were infected with pan-susceptible *M. tuberculosis*, suggesting that the diagnosis of tuberculosis was made too late to alter the fatal course of the infection. There is also a significant burden of undetected multidrug-resistant tuberculosis among HIV-coinfected individuals dying in this setting. New public health approaches that improve early diagnosis of tuberculosis and accelerate the initiation of treatment are urgently needed in this setting.

*Please see later in the article for the Editors' Summary*

## Abstract 2

A postmortem study by Ted Cohen and colleagues reveals a huge toll of tuberculosis among patients dying in hospitals in KwaZulu-Natal, South Africa.

## Editors' Summary

**Background:**

Every year, nearly 10 million people develop tuberculosis—a contagious bacterial infection that affects the lungs and other parts of the body—and nearly two million people die from the disease. Tuberculosis is caused by *Mycobacterium tuberculosis*, which spreads in airborne droplets when people with the disease cough or sneeze. Its characteristic symptoms are a persistent cough, weight loss, and night sweats. Diagnostic tests for tuberculosis include the microscopic examination of sputum samples (mucus brought up from the lungs by coughing) for *M. tuberculosis* bacilli, and mycobacterial culture (in which bacteriologists try to grow *M. tuberculosis* from sputum or tissue samples). Although tuberculosis can be cured by taking several powerful antibiotics regularly for at least 6 months, global efforts to control tuberculosis are being thwarted by the emergence of strains of *M. tuberculosis* that are resistant to several antibiotics (multidrug and extensively drug-resistant tuberculosis) and by the HIV epidemic; people who are infected with HIV, the virus that causes AIDS, are particularly susceptible to tuberculosis because of their weakened immune system.

**Why Was This Study Done?:**

In the past few years, tuberculosis has become the leading recorded cause of death in South Africa, a country where nearly a fifth of adults are infected with HIV. There are 122,000 recorded deaths from tuberculosis (including 94,000 deaths in HIV-positive people) in South Africa every year. However, because the accurate diagnosis of tuberculosis in HIV-positive people can be difficult—they are more likely to have sputum-negative tuberculosis than HIV-negative individuals—the true number of people dying because of tuberculosis is likely to be higher than the recorded number. Public-health experts in South Africa need an accurate picture of the tuberculosis deaths to help them improve tuberculosis control. In this postmortem study, the researchers determine the prevalence (the proportion of a population that has a disease) and drug sensitivity of tuberculosis among patients dying in a public hospital in KwaZulu-Natal, South Africa, to get a better estimate of how many people die because of tuberculosis in this setting.

**What Did the Researchers Do and Find?:**

The researchers collected respiratory tract secretions and lung, liver, and spleen samples from 240 adults who died in the Edendale public hospital in KwaZulu-Natal over a 10-month period in 2008/9. They looked for *M. tuberculosis* bacilli in the samples, tried to culture *M. tuberculosis* from them, and tested any bacteria that grew for antibiotic sensitivity. They also collected information on current tuberculosis treatment status, previous tuberculosis treatment, and HIV status for each deceased patient (decedent) from medical records and from relatives. Ninety-four percent of the decedents were HIV positive and 50% had culture-positive tuberculosis at the time of death. Of the 50% of the decedents who were being treated for tuberculosis, 58% were culture positive at the time of death. A similar percentage (42%) of the decedents who were not being treated for tuberculosis were culture positive at the time of death. Seventeen percent of all the positive cultures were multidrug resistant and 16% of patients dying during their first course of tuberculosis treatment were infected with multidrug-resistant bacteria. Seventy percent of decedents who remained culture positive despite receiving tuberculosis treatment were infected with *M. tuberculosis* strains that were susceptible to all antibiotics.

**What Do These Findings Mean?:**

These findings reveal the immense toll of tuberculosis among HIV-infected individuals in this hospital in KwaZulu-Natal. They show that many patients being treated for tuberculosis were culture positive at death despite being infected with antibiotic-sensitive *M. tuberculosis*, which suggests that diagnoses of tuberculosis are often made too late to alter the fatal course of infection. These findings also suggest that multidrug-resistant tuberculosis often goes undetected among HIV-infected individuals. Further studies are needed to confirm these findings elsewhere in South Africa and in other countries with a high HIV prevalence. Nevertheless, they suggest that public-health initiatives that improve the early diagnosis of tuberculosis, that introduce routine screening for tuberculosis among HIV-positive patients, and that accelerate the initiation of treatment for both tuberculosis and HIV might reduce the global death toll from tuberculosis.

**Additional Information:**

Please access these Web sites via the online version of this summary at http://dx.doi.org/10.1371/journal.pmed.1000296.

- The World Health Organization provides information on all aspects of tuberculosis, including information on tuberculosis and HIV, on tuberculosis in South Africa, and on the Stop TB Partnership (some information is in several languages)
- The US Centers for Disease Control and Prevention has information about tuberculosis and on tuberculosis and HIV coinfection
- The US National Institute of Allergy and Infectious Diseases also has detailed information on all aspects of tuberculosis
- Information is available from Avert, an international AIDS charity, on tuberculosis and HIV

## Introduction

The emergence of HIV in South Africa has resulted in a dramatic rise in the incidence of tuberculosis (TB). Between 1990 and 2007, as the adult HIV prevalence rose to 18.1% [1] the incidence of tuberculosis increased more than 3-fold from 301 to 948 cases/100,000 persons/year [2]. Approximately 80% of incident TB cases in South Africa are HIV-seropositive [2]. These coinfected individuals are more likely to have sputum smear–negative pulmonary or extrapulmonary TB [3],[4], which can be difficult to diagnose clinically. The World Health Organization recommends mycobacterial culture for patients diagnosed with sputum smear–negative tuberculosis. However, the utility of this investigation is limited by cost and long laboratory turn-around times.

The HIV epidemic has undermined TB control in South Africa, and the appearance of multidrug-resistant tuberculosis (MDRTB: resistant to the two most effective first-line drugs, isoniazid and rifampin) and extensively drug resistant tuberculosis (XDRTB: MDRTB with additional resistance to at least one fluoroquinolone and one injectable drug) has further compromised the effectiveness of standard interventions [5]. The interaction between drug-resistant TB and HIV was highlighted by an outbreak of nosocomial XDRTB among HIV-positive adults in Tugela Ferry, KwaZulu-Natal (KZN), South Africa; the outbreak was associated with a mortality rate of 98% and a median time of death at 16 days after diagnosis [6]. The diagnosis of MDRTB is laboratory-dependent and additional costs and time are both required for a definitive result.

TB has become the leading cause of death in South Africa by clinically determined death notification [7]; however, the precise role of TB in adult mortality is difficult to determine from death notifications due to the inherent clinical difficulties in securing an accurate diagnosis. In our study area, HIV seroprevalence is highest among adults in the 20- to 40-y age range, and the average life expectancy is 43 y [8]. In order to assess the magnitude of deaths associated with TB in young adults in KZN, we conducted a limited autopsy study of patients dying in a single public hospital between October 2008 and August 2009.

## Methods

### Ethics Statement

The biomedical research ethics committee at the University of KwaZulu-Natal and the KwaZulu-Natal Department of Health approved the protocol for this study.

### Study Setting

Edendale Hospital is an 860-inpatient-bed district and regional facility located near the city of Pietermaritzburg in the province of KwaZulu-Natal, South Africa. A previous study in this hospital found that 28% of admitted patients were diagnosed with active TB [9]. In 2006, the local incidence of TB was estimated at 1,094 cases/100,000 persons/year and the HIV prevalence among women in antenatal clinics was more than 39% [10],[11]. The total estimated number of prevalent MDRTB cases was 2,390 in 2006 and 2,799 in 2007 (unpublished data).

### Study Population

We recruited eligible decedents from a cohort of adult inpatients at Edendale until we reached our target sample size of 240. Eligibility criteria included age between 20 and 45 y at time of death, death occurring after admission to either the medical or surgical ward for inpatient treatment at Edendale Hospital, and the consent of a family member for inclusion in the study. Inpatients dying from trauma or obstetric complications were excluded.

A trained research nurse approached family members of recently deceased patients and, after informing them of the aims and protocol of the study, invited these families to participate. For logistical reasons, family members were approached between 08:00 and 14:00 three days a week. A detailed informed consent document was provided in both isiZulu and English. Family members were counseled on TB symptoms and how to access TB testing, and were encouraged to test for HIV infection in accordance with South African government guidelines. Close family contacts of decedents diagnosed with MDRTB were referred for specialist examination. HIV status of the decedent was not routinely reported to the family. Any close contact who signed a confidential form reporting a sexual relationship with the deceased was informed of the HIV status of the decedent. Family members were provided with compensation to defray the costs of travel to and from the hospital.

### Data Collection and Diagnostic Procedures

We collected demographic and medical history data from each participant's medical record. Information collected included age at time of death, date of hospital admission, inpatient wards occupied, inpatient medical diagnoses at time of death, current TB treatment status, history of TB antibiotic exposure (“new TB cases” are those with less than one month of previous exposure to TB drugs and “retreatment cases” are those with at least one month of previous TB treatment before the initiation of current TB therapy), other antibiotics received (including antiretroviral therapy), and known MDRTB exposure prior to admission or contact with a TB patient who had died within the previous 5 y.

The HIV status of decedents was collected from the medical record. For individuals without a documented HIV test result, a postmortem HIV test was done on blood or serum aspirated from the heart or great vessels, using a dried blood spot methodology [12]–[14]; accordingly, we were able to definitively classify the HIV status of every decedent included in the study.

A limited autopsy was performed to obtain specimens for microscopic examination for acid-fast bacilli and for *M. tuberculosis* culture. Respiratory tract secretions were obtained by saline lavage through a cricothyroid membrane puncture, and tissue was obtained by needle core biopsies of the lung, liver, and spleen. The liver and spleen were located using percussion and standard anatomical surface markings [15]. Lung biopsies were obtained through both second intercostal spaces, in the mid-clavicular line, with the needle directed posteriorly and inferiorly, and from areas that were dull to percussion. As greater amounts of tissue have been demonstrated to improve yield, we obtained multiple cores from each organ sampled [16]–[19]. If full-length core samples were successfully retrieved (at least 3 cm), then only three samples were taken, but if smaller samples were retrieved, up to five specimens were collected from each organ. Biopsy specimens were combined into a single pooled specimen. Samples were transported to the NHLS referral mycobacteriology laboratory at Inkosi Albert Luthuli Central Hospital. Standard decontamination procedures (using N-acetyl-L-cysteine and 4% sodium hydroxide with a final concentration of 1% for decontamination and exposure for 15–20 min) were used for mucolysis and to minimize contamination rates. Specimens were examined by fluorescent microscopy for acid-fast bacilli and cultured in liquid culture media (BACTEC MGIT 960 Becton Dickinson). Positive cultures were identified as *M. tuberculosis* using the niacin-nitrate test. Drug sensitivity testing for first- and second-line agents (isoniazid, rifampin, ethambutol, and streptomycin, kanamycin, ofloxacin) were performed on Middlebrook 7H10 solid media using standardized protocols [20],[21].

In order to evaluate the generalizability of our findings we reviewed medical records to assess the HIV seropositivity rate, the antiretroviral usage, and TB treatment status of a randomly sampled group of decedents who were eligible but not included in the study.

### Statistical Analysis

We used bivariate and multivariate logistic regression to identify independent associations between measured covariates and (1) the probability of TB and (2) the probability of MDRTB among those with TB at the time of death. We included all variables examined in the bivariate analysis in our multivariate models and also assessed whether or not antiretroviral therapy modified the association between HIV and each dependent variable. Associations with *p*-values <0.05 were considered statistically significant. Data were recorded on standard paper forms and entered into a Microsoft Access database and checked against original paper records by two different individuals. Analysis was done using Stata version 9.2 (College Station, TX, USA).

## Results

Two hundred and forty decedents were recruited into the study with dates of death beginning on 28 October 28 2008 and ending on 12 August 12 2009. Over this entire time period, a total of 997 deaths occurred among inpatients who were eligible for inclusion in the study. Table 1 displays selected characteristics of individuals included in this sample. We found no significant difference in the age and sex distribution of those included in the sample and those eligible to be in the sample. The median age was 33 y with an interquartile range (IQR) of 28–38 y for those actually included in the study and also 33 y (IQR 28–38 y) for those eligible to be in the study, *p* = 0.31; the sex distribution was 44% male in the study compared with 46% male among those eligible to be in the study) (*p* = 0.60). Two hundred and twenty six (94%) of the decedents in the study were HIV-positive; 200 of the seropositive individuals had been diagnosed with HIV before death. As such, the apparent HIV prevalence (not including those whose HIV infections were diagnosed after death) among those included in the study (200/240 = 83%) was not statistically significantly different than the observed HIV prevalence among a random selection of eligible decedents who were not included in the study (126/164 = 77%; *p* = 0.13). In total, 17% of HIV-positive decedents included in the study were receiving antiretroviral therapy at the time of death; of the subset of participants known to be HIV positive before autopsy, 20% were receiving antiretroviral drugs. Antiretroviral usage was lower among participants than among the random selection of known HIV positive decedents that were not included in the study (38/126 = 30%; p-value = 0.03). 15% of decedents had at least one previous hospitalization within the past 6 mo. Decedents died a median of 4 d (IQR 1–7 d) after admission to the hospital.

**Table 1:** Study population characteristics.

Category	Number
Total number of participants	240
Number of females (%) [n = 240]	134 (55.8%)
Median age in years (25%ile, 75%ile) [n = 240]	33 (28, 38)
Number HIV seropositive (% )[n = 240]	226 (94%)
Number of HIV-positive individuals receiving antiretrovirals (% among those seropositive)	39 (17%)
Number with a prior hospitalization within the last 6 months (%) [n = 204]	31 (15%)
TB treatment status [n = 237]	
Number currently on TB treatment, new case, initiation phase (%)	83 (35%)
Number currently on TB treatment, new case, continuation phase	20 (8%)
Number currently on TB treatment, retreatment case, initiation phase	13 (5%)
Number currently on TB treatment, retreatment case, continuation phase	2 (<1%)
Number currently on TB treatment, MDRTB treatment regimen	1 (<1%)
Number with previous TB diagnosis, not on TB therapy at time of death	16 (7%)
Number with suspected TB, not on TB therapy at time of death	16 (7%)
No previous diagnosis of TB	85 (36%)
Number with known exposure to MDR [n = 70]	3 (4%)
Number with a recent death within the home [n = 221]	15 (7%)
Number with history of failed TB treatment [n = 199]	8 (4%)

One hundred and nineteen decedents (50%) were being treated for TB in accordance with national guidelines at the time of death; this was not statistically significantly different than the proportion receiving tuberculosis treatment among the random selection of eligible decedents that were not included in the study (77/164 = 47%; *p* = 0.55). Sixty-one percent of patients on TB treatment were diagnosed during their final hospital admission. The median length of hospitalization before death did not differ significantly (*p* = 0.85) between those diagnosed with TB and started on anti-TB drugs during this final admission (a median of 5 d between hospitalization and death, IQR 1–9 d) and those suspected of having TB but were not started on anti-TB regimens and were found to be culture positive only after death (a median of 4 d between hospitalization and death, IQR 3–6 d). Fewer than 10% of decedents had either known exposures to MDRTB, recent unexplained deaths among household contacts, or a history of previous failed TB treatment.

### Culture-Positive Tuberculosis Disease

A total of 236 specimens were examined in the laboratory; of these, 11 (4.7%) were contaminated; this is consistent with expected laboratory performance [19]. Smear examination of autopsy specimens was positive in 79 (34%) of those tested, and 110 (50%) were culture-positive for *M. tuberculosis* (Table 2). Three specimens (<3%) were shown to be non-tuberculous mycobacteria.

**Table 2:** Postmortem smear and culture status among participants, categorized by TB treatment status.

Smear/Culture Status	On TB Treatment at Time of Death [n = 117] (50%)	Not on TB Treatment at Time of Death [n = 119] (50%)
Overall smear positive	39% [46/117]	29% [33/115]
Overall culture positive	58% [64/111]	42% [46/110]

	New, Initiation [n = 81]	New, Continuation [n = 20]	Retreatment, Initiation [n = 13]	Retreatment, Continuation [n = 2]	MDR Regimen [n = 1]	Suspect TB, not on treatment [n = 15]	Tuberculosis not suspected [n = 100]
Smear positive	44% [36/81]	15% [3/20]	38% [5/13]	50% [1/2]	100% [1/1]	53% [8/15]	25% [25/100]
Culture positive	65% [51/78]	26% [5/19]	55% [6/11]	50% [1/2]	100% [1/1]	43% [6/14]	42% [40/96]

Among decedents receiving or not receiving antitubercular therapy, 64 (58%) and 46 (45%) were culture positive, respectively. Six (43%) TB suspects who were not yet on treatment had a positive culture. Among those participants in whom TB was not suspected at the time of death, 40 (42%) were culture positive; 39 of these patients (98%) were HIV seropositive. The top three leading inpatient diagnoses for these unsuspected *M. tuberculosis* culture-positive individuals were: pneumonia/lower respiratory infection (25%), meningitis (18%), and gastroenteritis (15%).

In bivariate analysis, individuals receiving TB treatment and males were significantly over-represented among those with positive cultures at time of death (Table 3). No other measured factors were associated with culture positivity. Among the decedents who were positive for HIV, antiretroviral use did not modify the probability of TB. Adjusting for each of the other factors, males had nearly twice the odds of having a positive culture for *M. tuberculosis* than females (odds ratio [OR] 1.98; 95% confidence interval [CI] 1.12–3.52) and individuals on TB treatment at the time of death had over 80% increased odds of culture positivity (OR 1.82; 95% CI 1.05–3.18).

**Table 3:** Bivariate and multivariate association with culture positive TB at time of death.

Category	Unadjusted OR (95% CI)	*p*-Value	Adjusted OR (95% CI)	*p*-Value
Male	2.07 (1.21–3.54)	<0.01	1.98 (1.12–3.52)	0.02
Age (for each additional year of age)	1.00 (0.96–1.03)	0.84	0.99 (0.95–1.03)	0.69
HIV positive	2.27 (0.68–7.60)	0.18	3.03 (0.85–10.74)	0.09
Antiretroviral use (among those with HIV)	0.66 (0.32–1.35)	0.26	0.63 (0.30–1.33)	0.22
Recent hospitalization (within previous 6 months)	1.04 ( 0.96–1.13)	0.34	1.06 (0.97–1.17)	0.18
On TB treatment at the time of death	1.84 (1.11–3.23)	0.02	1.82 (1.05–3.18)	0.03

### Drug Resistance

Seventeen (17%) of the *M. tuberculosis* isolates were MDR. Only one XDR isolate was identified; it was found in an HIV-positive patient with no recent history of hospitalization who was in the initiation phase of his first course of TB chemotherapy. Only one specimen was monoresistant to isoniazid and one monoresistant to rifampin; all other samples that were resistant to one of these drugs were resistant to both drugs. Table 4 displays resistance profiles categorized by TB treatment status.

**Table 4:** Resistance profiles for those with positive cultures, categorized by TB treatment status.

Resistance Profile	On TB Treatment at Time of Death and Culture Positive					Not on TB Treatment at Time of Death and Culture Positive		
	New, Initiation [n = 50]	New, Continuation [n = 5]	Retreatment, Initiation [n = 5]	Retreatment, Continuation [n = 1]	MDR Regimen [n = 1]	Previous TB, not on treatment [n = 4]	Suspect TB, not on treatment [n = 6]	No previous TB diagnosis [n = 32]
Pan-sensitive	78% [38/49]	20% [1/5]	80% [4/5]	0% [0/1]	0% [0/1]	75% [3/4]	83% [5/6]	91% [29/32]
INH-R^a^	18% [9/49]	80% [4/5]	20% [1/5]	100% [1/1]	100% [1/1]	25% [1/4]	17% [1/6]	3% [1/32]
RIF-R^a^	23% [10/49]	80% [4/5]	0% [1/5]	100% [1/1]	100% [1/1]	25% [1/4]	0% [0/6]	3% [1/32]
STR-R^a^	10% [5/49]	60% [3/5]	0% [1/5]	100% [1/1]	100% [1/1]	0% [0/4]	0% [0/6]	9% [3/32]
ETH-R^a^	2% [1/50]	0% [0/5]	20% [1/5]	0% [0/1]	0% [0/1]	0% [0/4]	0% [0/6]	0% [0/32]
KAN-R^a^	2% [1/50]	0% [0/5]	0% [0/5]	0% [0/1]	0% [0/1]	0% [0/4]	0% [0/6]	0% [0/32]
OFX-R^a^	2% [1/50]	0% [0/5]	20% [1/5]	0% [0/1]	0% [0/1]	0% [0/4]	0% [0/6]	0% [0/32]
MDR^b^	16% [8/49]	80% [4/5]	20% [1/5]	100% [1/1]	100% [1/1]	25% [1/4]	0% [0/6]	3% [1/32]
XDR	1	0	0	0	0	0	0	0
MOTT	1	0	1	0	0	0	0	1

a Alone or in combination with any other resistance.

b Among those with sufficient DST results to classify MDR – does not include XDR.

Bivariate analyses revealed that a recent previous hospitalization and current tuberculosis therapy were each associated with an increased probability of detecting MDR among those with disseminated *M. tuberculosis* (Table 5). Multivariate analyses revealed that among those who were culture positive, being on current TB treatment was associated with a greater than 6-fold increase in the odds of MDR (OR 6.56; 95% CI 1.31–32.75). Adjusting for other factors, recent hospitalization did not remain statistically significantly associated with MDR (OR 1.16; 95% confidence interval 0.99–1.36).

**Table 5:** Bivariate and multivariate associations with MDR (among those that were culture positive and with sufficient DST) at time of death.

Category	Unadjusted OR (95% CI)	*p*-Value	Adjusted OR (95% CI)	*p*-Value
Male	0.45(0.15–1.33)	0.15	0.53 (0.14–2.00)	0.35
Age (for each additional year of age)	0.92 (0.84–1.01)	0.07	0.94 (0.85–1.04)	0.21
HIV positive	0.38 (0.33–4.46)	0.44	0.55 (0.04–7.99)	0.66
Antiretroviral use (among those with HIV)	2.63 (0.71–9.82)	0.15	1.81 (0.38–8.50)	0.29
Recent hospitalization (within previous 6 months)	1.15 (1.00–1.32)	0.05	1.16 (0.99–1.36)	0.06
On TB treatment at the time of death	6.52 (1.41–30.27)	0.02	6.56 (1.31–32.75)	0.01

## Discussion

While TB is recognized as a major cause of early death in KwaZulu-Natal, South Africa, our findings reveal that the toll of TB is far larger than has been previously reported. Despite efforts to prioritize diagnosis and treatment of TB, especially among those coinfected with HIV, we found that half of the inpatients who died at a single hospital had evidence of TB at the time of death. Although almost half of those who died were on TB treatment at the time of death, more than half of those on treatment still had evidence of viable *M. tuberculosis* in their postmortem specimens. Nearly half of the decedents who were not on TB treatment at the time of death also had evidence of TB. Since needle core biopsies of organs and respiratory specimens are unlikely to detect all cases of disease, the proportion of decedents from which we could grow *M. tuberculosis* should be considered a minimum estimate of the actual fraction with TB at the time of death.

Our data are consistent with a growing body of literature that shows extraordinarily high rates of TB in young HIV-positive adults dying in African hospitals [22]–[24]. Although we did not preferentially target HIV-positive individuals for inclusion, nearly all (94%) of the decedents who were eligible and entered our study were HIV seropositive. By the time this study had begun, more than 7,000 adults had initiated antiretroviral therapy at Edendale Hospital. Antiretroviral use was not associated with the probability of culture-positive TB in our study population; however, we were not able to determine the duration of antiretroviral therapy prior to death.

Since we cultured all samples collected, we were able to perform speciation and assess the drug sensitivity of the *M. tuberculosis* isolates that we detected. Forty-three (70%) culture positive patients who died while receiving TB treatment had pan-sensitive *M. tuberculosis* isolated. This suggests either that the diagnosis of TB was made too late to alter the fatal course of the infection, that adherence was not adequate, or that drug malabsorption [25] played some role in these poor outcomes among patients who would otherwise be expected to respond well to standard TB treatment [26],[27].

Although previous hospitalization was not found to be independently associated with an increased risk of MDR, the association almost reached the level of statistical significance (*p* = 0.06). If there is a relationship between previous hospitalization and risk of dying with MDR, this could be consistent with either a longer course of TB (either causing resistance or as a result of resistance) or nosocomial transmission of resistant disease. An increased risk of MDR among those on first-line TB treatment may indicate either an increased risk of acquired resistance or that individuals with MDR are more likely to remain culture positive despite this treatment. That nearly one in six TB patients dying during the initiation phase of a first TB treatment regimen were MDR provides support for the latter hypothesis.

It is startling that 17% of the *M. tuberculosis* isolates we detected were MDR and that more than 16% of patients dying during the initiation phase of their first ever course of TB therapy have MDRTB. These patients are quite likely to have been primarily infected with drug-resistant *M. tuberculosis* and, since many had no recent history of hospitalization, they provide additional evidence of community transmission of drug-resistant TB [6]. The scarcity of XDRTB in our data confirms that the Tugela Ferry outbreak has not extended to the Edendale Hospital catchment area. Nevertheless, these findings point to a hidden burden of MDR disease among HIV-coinfected individuals in KZN, and the contribution of MDRTB to early mortality in this highly vulnerable population must be further investigated.

This study has several limitations. We obtained the clinical data and epidemiological data on decedents from two sources: (1) their medical charts and (2) interviews with family members. While the medical charts were the most comprehensive clinical record available, these data were not collected prospectively for the purpose of the study. The accuracy of information obtained from family members about potential TB exposures within the home may not be reliable. Because of cost and human resource concerns, we pooled all specimens collected from each participant for culture; this limits our ability to identify the site of disease involvement among those with culture-positive TB. We also did not have resources to carry out additional histological examination of tissues gathered at autopsy; previous studies [22]–[25] have shown that additional diagnoses would likely have been made in this population that is highly exposed to and possibly infected with multiple opportunistic pathogens.

The prevalence of TB detected in our study should serve as an alarm call in this setting of high HIV prevalence, antiretroviral scale-up, and the emergence of MDR and XDRTB. The fact that nearly half of all cases included in our survey had culture-positive TB at the time of death, and that this proportion was essentially the same regardless of TB treatment status or antiretroviral use among those with HIV infection, mandates the implementation of early, more efficacious interventions against TB and HIV. Decreasing patient and provider delays in the diagnosis of TB in this high HIV burden setting is essential to avert unnecessary early deaths [28],[29]. Physicians caring for HIV-positive or status-unknown patients should be aware that TB may occur together with other, more acute conditions. Symptom screening, physical examination, and evaluation of the chest radiograph for active TB is essential [30]. Acutely ill hospitalized patients should routinely submit sputum for TB screening with laboratory-based approaches that increase the rapidity and/or sensitivity of diagnosis of TB such as front-loaded (same day) smear microscopy [31], sputum concentration methods [32], and LED fluorescence microscopy [33]. Antiretroviral therapy prevents TB ; however, less than a fifth of the HIV-positive decedents in this study had started treatment. Expanded access to, and timely initiation of, antiretroviral therapy is essential in order to reduce TB mortality [5],[34].

The substantial proportion of patients dying with MDRTB during the initiation phase of their first course of TB therapy highlights the difficulty of selecting appropriate treatment in locations where drug resistance is emerging, even among those patients for whom a diagnosis of TB is actually made. Promising molecular tools for the rapid diagnosis of drug-resistant TB have been developed [35],[36], and their adoption in these settings has already begun [37]. The support of the Stop TB Partnership for field evaluation of a line probe assay for MDRTB is a significant advance. However, the assay is recommended only for sputum smear-positive or culture-positive specimens, which limits usage in resource-limited high-HIV prevalent settings such as KZN. The availability of rapid and accurate drug resistance testing must also be accompanied by expansion of access to second-line drugs and support to enable these patients to adhere to the lengthy treatment regimens that are required to treat highly drug resistant TB.

The large burden of unrecognized disease among hospitalized patients also emphasizes the need to improve infection control both within nosocomial settings and within the community in locations where transmission is likely to occur. Efforts to increase the availability of TB screening for those with HIV and to conduct intensified case finding will have the dual benefit of getting individuals more quickly onto appropriate therapy and reducing the opportunity for onward transmission of TB.

## Abbreviations

CI: confidence interval
IQR: interquartile range
KZN: KwaZulu-Natal
MDR: multidrug resistant
OR: odds ratio
TB: tuberculosis
XDR: extensively drug resistant
